# A community-based One Health approach to the pandemic dynamics of *Escherichia coli* ST131

**DOI:** 10.1128/aac.00007-26

**Published:** 2026-06-23

**Authors:** Aiko Maeda, Toyotaka Sato, Akira Fukuda, Torahiko Okubo, Masaru Usui, Mana Tohyama, Jirachaya Toyting-Hiraishi, Naoyuki Tsukamoto, Akio Suzuki, Yuuji Hoshino, Shingo Torigoe, Keiichiro Sakakibara, Satoshi Tamai, Tooru Tachibana, Satoshi Takahashi, Shin-ichi Yokota, Motohiro Horiuchi

**Affiliations:** 1 Laboratory of Veterinary Hygiene, Faculty of Veterinary Medicine, Hokkaido University, Sapporo, Japan; 2 Graduate School of Infectious Diseases, Hokkaido University 12810, Sapporo, Japan; 3 One Health Research Center, Hokkaido University 12810, Sapporo, Japan; 4 Veterinary Research Unit, International Institute for Zoonosis Control, Hokkaido University, Sapporo, Japan; 5 Food Microbiology and Food Safety Unit, Division of Preventive Veterinary Medicine, School of Veterinary Medicine, Rakuno Gakuen University 13022, Ebetsu, Japan; 6 Faculty of Health Sciences, Hokkaido University 12810, Sapporo, Japan; 7 Sapporo Mirai Laboratory, Sapporo, Japan; 8 Kikusui Small Animal Hospital, Sapporo, Japan; 9 Ainosato Animal Hospital, Sapporo, Japan; 10 North Animal Clinic, Sapporo, Japan; 11 Anicom Specialty Medical Institute Inc., Tokyo, Japan; 12 Hokko Animal Hospital, Sapporo, Japan; 13 Department of Infection Control and Laboratory Medicine, Sapporo Medical University School of Medicine 92187 , Sapporo, Japan; 14 Division of Laboratory Medicine, Sapporo Medical University Hospital 666333, Sapporo, Japan; 15 Division of Microbiology, Department of Infectious Diseases, Sapporo Medical University School of Medicine, Sapporo, Japan; Universita degli studi di Roma La Sapienza, Rome, Italy

**Keywords:** *Escherichia coli*, fluoroquinolone, ST131, antimicrobial resistance, One Health

## Abstract

Large-scale genomic studies have advanced our understanding of the global dissemination of antimicrobial-resistant (AMR) bacteria, yet most have been limited by inconsistent sampling periods, heterogeneous geographic coverage, and weak epidemiological linkages among isolates. Consequently, the fine-scale processes underlying the establishment and maintenance of AMR pandemics remain unclear. To address these limitations, we established a rigorously controlled community- and household-integrated One Health framework that incorporates human, animal, and environmental reservoirs within a single urban area and unified sampling period, focusing on the pandemic fluoroquinolone-resistant (FQ-R) *Escherichia coli* sequence type (ST) 131. Using this model, we analyzed FQ-R *E. coli* isolates collected from humans, companion animals, sewage, and livestock in the Sapporo area, Japan, during 2021–2022. Among 2,358 *E. coli* isolates, 235 FQ-R isolates, including 88 ST131 isolates, underwent genomic analyses based on pan-, accessory-, and core genome data. These analyses revealed high clonal diversity but clear population structuring within ST131, with several subclusters shared between human and companion animal sources. High-resolution core genome SNP analysis delineated community-level transmission dynamics, demonstrating ST131 carriage among healthy humans and suggesting asymptomatic human-to-human spread and household-level human–companion animal transmission. These carriage isolates harbored multiple AMR genes and virulence factors and exhibited high genetic similarity to clinical isolates, indicating potential contributions to hospital-associated infections. Phylogenetic integration with international ST131 genomes demonstrated that transmission events in Sapporo are embedded within the broader global expansion of ST131. This study provides a scalable model for resolving local-to-global AMR dissemination and underscores the need for community- and household-level interventions within a One Health framework.

## INTRODUCTION

Antimicrobial-resistant (AMR) bacteria have become a major global health concern since the 2000s. Resistant pathogens often render standard antibiotic therapies ineffective, resulting in increased morbidity and mortality. Recognizing the scale of the problem, the World Health Organization (WHO) adopted a Global Action Plan on AMR in 2015 (1). Since then, many countries have developed national action plans, accelerating research and surveillance efforts. In the human healthcare sector, AMR-related mortality has been quantified with increasing precision. According to the latest study, an estimated 4.71 million deaths were associated with AMR in 2021 (2). The AMR-associated deaths are projected to rise to 8.22 million annually, resulting in a total of 169 million deaths between 2025 and 2050. Notably, these trends show substantial regional variability, underscoring the need for localized, context-sensitive interventions.

Among Gram-negative bacteria, *Escherichia coli* accounts for the highest number of AMR-associated deaths (2). Within *E. coli*, fluoroquinolone-resistant (FQ-R) and third-generation cephalosporin-resistant (3GC-R) strains are identified as major contributors to AMR burden (3). Importantly, 3GC-R *E. coli* was ranked second out of 24 pathogens on the WHO’s Bacterial Priority Pathogens List (BPPL) in 2024, up from fourth place in 2017 (4). Notably, it is well-known that extraintestinal pathogenic *E. coli* (ExPEC) frequently exhibit co-resistance to FQs and 3GCs (5, 6), reflecting the increasing global concern despite ongoing AMR countermeasures, as FQs are also widely used antimicrobial agents for *E. coli* infections (7).

In ExPEC, a particular FQ-R *E. coli* lineage, sequence type (ST) 131, has achieved pandemic spread in clinical settings, often exhibiting 3GC-R and causing serious bloodstream and urinary tract infections in humans (8, 9). Consequently, ST131 is widely recognized as an “international high-risk clone” or “pandemic clone” (6, 10–12). Thus, focusing on and monitoring of FQ-R *E. coli* is essential to understand the mechanisms underlying the establishment of AMR pandemic with 3GC-R. Beyond clinical cases, ST131 has also been isolated from healthy humans, companion animals, livestock, wildlife, and environmental sources such as river and lake water (12). Studies suggest that contaminated food, wastewater, and migratory birds may facilitate its community-level transmission (13–15). Supporting this, several studies have reported close genetic relatedness between human-derived ST131 strains and those from companion animals, surface water, and wild animals (14, 16, 17). Moreover, transmission of ST131 between companion animals and household members has been reported (18–20). These findings highlight the necessity of cross-sectoral molecular epidemiological studies based on the One Health approach (21).

Pandemics emerge through the accumulation and expansion of transmission events within communities. Thus, understanding AMR dissemination in community settings is essential for elucidating the mechanisms underlying bacterial pandemics. Although numerous molecular epidemiological studies of ST131 have been conducted globally, major gaps remain regarding how ST131 spreads within communities. Limitations stem primarily from two issues: (i) studies with large isolate numbers often used heterogeneous regions sampling areas and/or time periods, weakening epidemiological linkage (5, 21–23) and (ii) studies examining well-defined epidemiological contexts (such as patient-to-patient transmission or owner–pet relationships) typically analyzed too few isolates for comprehensive interpretation (20, 24–26).

To clarify the mechanisms underlying the establishment of the ST131 pandemic, more robust investigations addressing limitations (i) and (ii) simultaneously are required. Such studies should employ a standardized One Health-based design and analyze sufficient isolates collected from defined community settings during a fixed observation period. However, no comprehensive study has yet examined the dissemination of ST131 within a single community under unified sampling conditions.

In this study, we collected *E. coli* isolates from patients (from a university hospital and community clinics), healthy humans, companion animals (dogs and cats), their pet owners, environmental water sources (sewage and rivers), and livestock (cattle, pigs, and chickens) in the Sapporo area, Japan, during 2021–2022. We then examined their genetic relatedness as a comprehensive model to investigate the dissemination of FQ-R *E. coli* within a community.

## RESULTS

### Overview of *E. coli* isolates and FQ-R across multiple sources in the Sapporo area

In this study, we collected a total of 2,358 *E. coli* isolates from diverse sources in the Sapporo area in Japan during 2021–2022. The sampling sources included humans (university hospital patients, community clinic patients, and healthy humans), companion animals (dogs and cats), livestock (cattle, pigs, and chickens), and sewage and river water. Clinical specimens from university hospital patients mainly consisted of urine samples, suggesting that these isolates were likely associated with urinary tract infections and, therefore, represented disease-causing *E. coli*. In contrast, fecal samples were collected from patients in community clinics to assess the extent to which FQ-R *E. coli* isolates are introduced into community healthcare settings. Therefore, the FQ-R *E. coli* isolates from these patients were not considered causative agents of disease but were primarily regarded as colonizing strains. Fecal samples were also collected from healthy humans. To evaluate the carriage and dissemination of FQ-R *E. coli* to the human society, rectal swab samples were collected from companion animals and livestock. Additionally, sewage and river water samples were obtained from both central and peripheral areas of Sapporo.

Based on the susceptibility testing to ciprofloxacin, 220 *E. coli* isolates were identified as FQ-R. The overall FQ-R rate was 9.3% (*n* = 220/2,358). The proportions by source were as follows: 14.7% (*n* = 96/651) in humans [17.2% (*n* = 37/215) in university hospital patients, 12.7% (*n* = 28/220) in patients from community clinics, and 14.4% (*n* = 31/216) in healthy humans], 26.2% (*n* = 51/195) in companion animals, 12.8% (*n* = 49/384) in sewage, 0.9% (*n* = 6/672) in river water, and 3.9% (*n* = 18/456) in livestock [4.2% (*n* = 13/306) in cattle, 0.0% (*n* = 0/75) in pigs, and 6.7% (*n* = 5/75) in chickens]. The overall prevalence of ST131 was 3.3% (*n* = 77/2,358). These isolates were derived from university hospital patients, community clinic patients, healthy humans, companion animals, and sewage (Table 1). Overall differences in both FQ-R rates and the proportion of ST131 among FQ-R isolates across sources were highly significant (*P* < 0.001). Notably, the FQ-R rate in companion animals was significantly higher than that in healthy humans, whereas the rates in river water and livestock were significantly lower. Furthermore, regarding the proportion of ST131 among FQ-R isolates, there was no significant difference between companion animals and healthy humans, but the proportions in livestock and river water were significantly lower.

**TABLE 1:** Distribution of FQ-R *E. coli* and ST131 isolates derived from multiple sources^*a*^

Source	Total no. of *E. coli* isolates	No. of FQ-R isolates^*b*^			No. of FQ-S isolates^*c*^
		Total	ST131	Non-ST131	
Patient/university	215	37 (17.2%)	23 (10.7%)	14 (6.5%)	178 (82.8%)
Patient/community	220	28 (12.7%)	13 (5.9%)	15 (6.8%)	192 (87.3%)
Healthy humans	216	31 (14.4%)	12 (5.6%)	19 (8.8%)	185 (85.6%)
Companion animals	195	51 (26.2%)^*^	21 (10.8%)	30 (15.4%)	144 (73.8%)
Sewage	384	49 (12.8%)	8 (2.1%)	41 (10.7%)	335 (87.2%)
River water	672	6 (0.9%)^***^	0 (0.0%)	6 (0.9%)	666 (99.1%)
Livestock	456	18 (3.9%)^***^	0 (0.0%)^††^	18 (3.9%)	438 (96.1%)
Total	2,358	220 (9.3%)	77 (3.3%)	143 (6.1%)	2,138 (90.7%)

^*a*^ Values show number of isolates. Statistical significance relative to healthy humans is indicated by asterisks for FQ-R rates (*: Padj < 0.05, ***: Padj < 0.001) and by daggers for ST131 distribution (^††^: Padj < 0.01). *P*-values were calculated using the χ test for overall FQ-R rates and Fisher’s exact test with Monte Carlo simulation for ST131 distribution, followed by Benjamini-Hochberg FDR correction for pairwise post-hoc comparisons.

^*b*^ FQ-R, fluoroquinolone resistant.

^*c*^ FQ-S, fluoroquinolone susceptible.

To more efficiently isolate FQ-R *E. coli* from companion animals and livestock, we additionally used ciprofloxacin-supplemented agar plates. Using this approach, we successfully recovered FQ-R *E. coli*, from companion animals (*n* = 51) and from livestock (*n* = 69; 27 from cattle, 3 from pigs, and 39 from chickens). After excluding duplicate clones obtained from the same animals, a total of 235 non-redundant FQ-R *E. coli* isolates were included in the subsequent whole-genome sequencing (WGS) analyses.

### Identification and prevalence of FQ-R *E. coli* among sequenced isolates

WGS was performed on FQ-R *E. coli* isolates. In total, 136 FQ-R *E. coli* isolates were analyzed, 96 from human (37 from university hospital patients, 28 from community clinic patients, and 31 from healthy humans), 17 from companion animals, and 23 from livestock (10 from cattle, 1 from a pig, and 12 from chickens). In addition, all FQ-R isolates from sewage (*n* = 49) and river water (*n* = 6) were included, as these environments are influenced not by a single source but by contamination from multiple ones, primarily associated with human activities. Furthermore, 38 previously reported isolates from companion animals and 6 isolates from companion animal owners (2 obtained in this study and 4 previously reported) collected during the same period were also included (20, 27). These 6 isolates from owners were incorporated into the healthy human group. Consequently, a total of 235 isolates were subjected to WGS analysis (Table S1).

**Fig 1:**
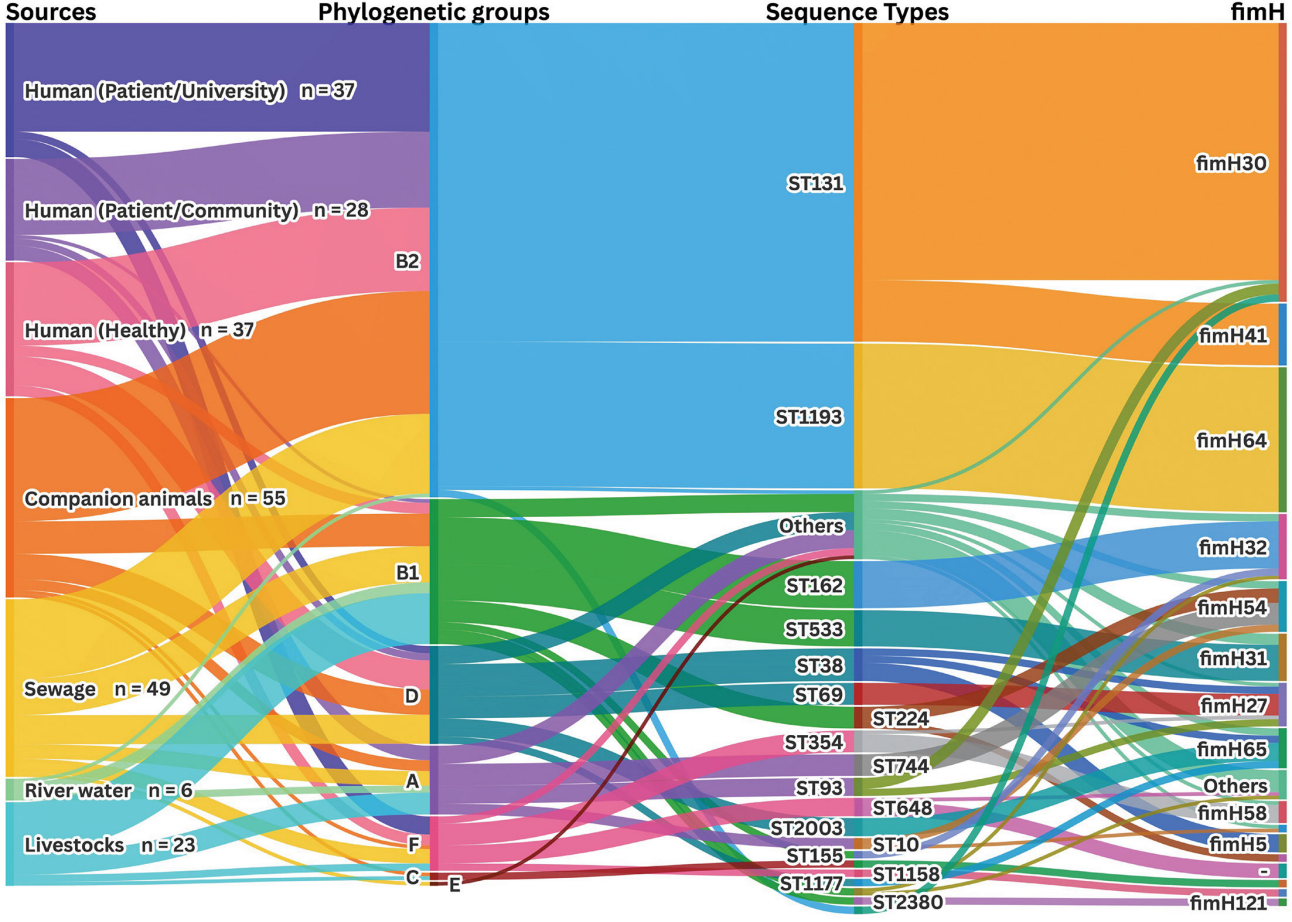
Distribution of FQ-R *E. coli* isolates across sources, phylogenetic groups, STs, and *fimH* alleles. Flow width is proportional to the number of isolates (*n*).

WGS analysis revealed that phylogenetic group B2 was predominant, accounting for 55.7% (131/235 isolates) of FQ-R *E. coli* isolates. These were mainly derived from humans, companion animals, and sewage and were absent from livestock (Fig. 1 and Fig. S1a). In contrast, phylogenetic group B1 represented 17.0% (40/235) of isolates. The B1 isolates were not recovered from university hospital patients, whereas they were more frequently observed in livestock and river water.

Within phylogenetic group B2, 67.2% (88/131) and 30.5% (40/131) of isolates were identified as ST131 and ST1193, respectively (Fig. 1). All 88 ST131 isolates belonged to either two *fimH* types, *fimH*30 (80.7%, *n* = 71) or *fimH*41 (19.3%, *n* = 17) (Fig. 1). In the classification of ST131 clades, isolates with *fimH*30 corresponded to clades C0, C1 (C1-M27 and C1-nM27), and C2, whereas isolates with *fimH*41 corresponded to clade A and unclassified lineages. In contrast to ST131, all 40 ST1193 isolates possessed a single *fimH* type, *fimH*64. ST131 and ST1193 were predominant in humans, companion animal, and sewage, whereas no ST131 isolates were identified from river water and livestock samples (Fig. S1b). ST131 was the most prevalent FQ-R *E. coli* lineage across human and companion animal samples, accounting for 62.2%, 46.4%, 40.5%, and 52.7% of isolates from university hospital patients, community clinic patients, healthy humans, and companion animals, respectively. ST1193 was the second most common FQ-R *E. coli* lineage in university hospital patients (16.2%), community clinic patients (21.4%), healthy humans (21.6%), and companion animals (9.1%). In sewage, ST1193 was the most prevalent (28.6%), followed by ST131 (16.3%).

Among ST131 isolates, clade C1 was the most frequent, accounting for 68.2% (*n* = 60/88), comprising 37.5% (*n* = 33) C1-nM27 and 30.7% (*n* = 27) C1-M27 isolates. The predominance of clade C1 was observed in isolates from university hospital patients (52.2%), community clinics (69.2%), healthy humans (86.7%), companion animals (72.4%), and sewage (62.5%) (Fig. S1c).

Clade A was the second most frequent ST131 clade, accounting for 13.6% (*n* = 12/88). It was detected in university hospital patients (13.0%), community clinic patients (15.4%), healthy humans (6.7%), companion animals (13.8%), and sewage (25.0%). Clade C2 accounted for 10.2% (*n* = 9/88) of isolates and was identified in university hospital patients (21.7%), community clinic patients (15.4%), healthy humans (6.7%), and companion animals (3.4%), but not in sewage. Clade C0 (2.3%, *n* = 2/88) was isolated only from university hospital patients (8.7%). Unclassified ST131 isolates (5.7%, *n* = 5/88) were detected in university hospital patients (4.3%), companion animals (10.3%), and sewage (12.5%).

Within phylogenetic group B1, ST162 (32.5%, *n* = 13/40) and ST533 (25.0%, *n* = 10/40) were the predominant lineages. ST162 isolates originated from sewage (38.5%, *n* = 5/13), humans and companion animals (23.1%, each *n* = 3/13), and river water (15.4%, *n* = 2/13) (Fig. 1 and Fig. S1b). ST533 was exclusively derived from livestock (all from chicken). In river water, the phylogenetic group B1 and ST162 was the most frequently isolated, followed by ST1193.

### Pangenome and clusters of orthologous groups functional categories of FQ-R *E. coli* isolates

**Fig 2:**
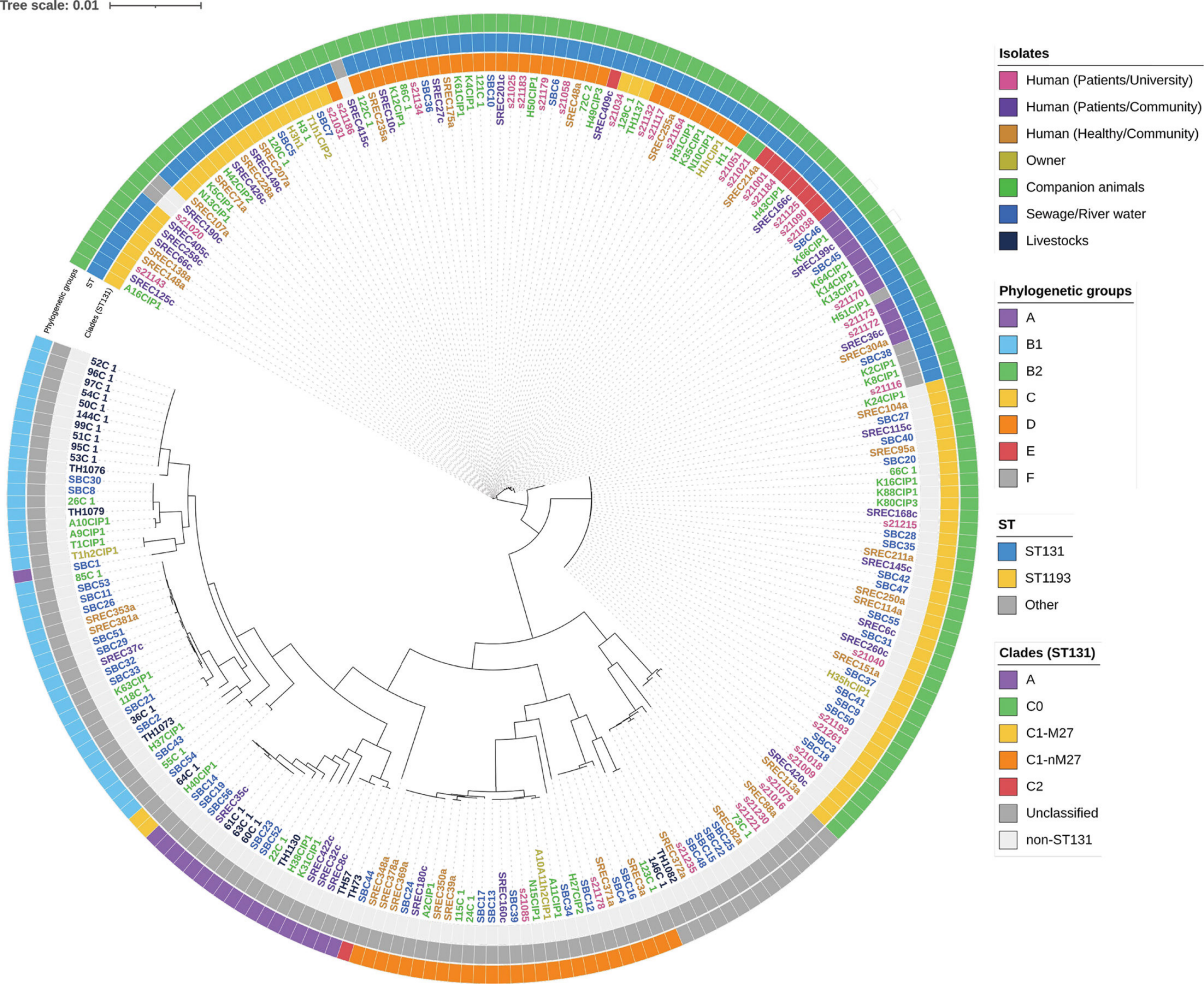
Pan-genome overview of 235 FQ-R *E. coli* isolates. The circular phylogenetic tree is based on the pangenome generated by Roary.

To characterize the genomic features of the major pandemic lineages ST131 and ST1193 in the Sapporo area and to assess their genetic relationships across multiple sources, we performed pangenome and COG functional category analyses as an initial step toward identifying key sources for detailed genomic investigation. Pangenome analysis revealed that the 235 FQ-R *E. coli* isolates shared 2,648 core genes (present in all isolates), while the accessory genome (present in <99% of isolates) consisted of 22,927 genes. A circular phylogenetic tree constructed from the pangenome analysis was annotated with phylogenetic groups and STs (Fig. 2). The phylogenetic tree revealed multiple clades corresponding to known phylogroups, including B2, B1, D, A, F, C, and E. Among them, phylogenetic group B2 exhibited a clear hierarchical structure, characterized by internal clustering of isolates by ST, such as ST131 and ST1193.

Of the 22,927 accessory genes identified, 18,585 genes (81.1%) were annotated with COG categories, while the remaining 4,342 genes (18.9%) were classified as “Unannotated.” To elucidate the functional gene repertoires of the major FQ-R *E. coli* clones in the Sapporo area, we compared their distributions of Clusters of Orthologous Groups (COG) categories (Fig. S2 and Table S2).

First, we compared the numbers of unique accessory genes in ST131 and ST1193 with those in non-ST131 or non-ST1193 *E. coli* populations (Other strains). The numbers of unique accessory genes in ST131 and ST1193 were significantly higher in several COG categories compared with other strains. These included categories related to cellular processes and signaling—M (cell wall/membrane/envelope biogenesis), N (cell motility), and V (defense mechanisms); metabolism/poorly characterized—G (carbohydrate transport and metabolism), P (inorganic ion transport and metabolism), and S (function unknown); and information storage and processing—K (transcription) and L (replication, recombination, and repair).

Next, we performed enrichment analysis to assess the functional composition of these categories relative to other strains. This analysis revealed that category N (Odds Ratio [OR] = 4.99, adjusted *P*-values; Padj = 5.4 × 10^−19^ in ST131; OR = 4.96, Padj = 1.1 × 10^−19^ in ST1193) was significantly enriched as specific functional compositions in both ST131 and ST1193 among the categories with significant numbers of unique accessory genes shown in Fig. S2 (Fig. S3a). Finally, we directly compared ST131 and ST1193. Although no significant difference was observed in the total number of unique accessory genes between them, enrichment analysis of category I (lipid transport and metabolism), revealed that ST131 possessed functionally distinct genes compared with ST1193 (Fig. S3b and Table S2).

### Accessory genome analysis and AMR/virulence factor profiling of ST131 isolates

**Fig 3:**
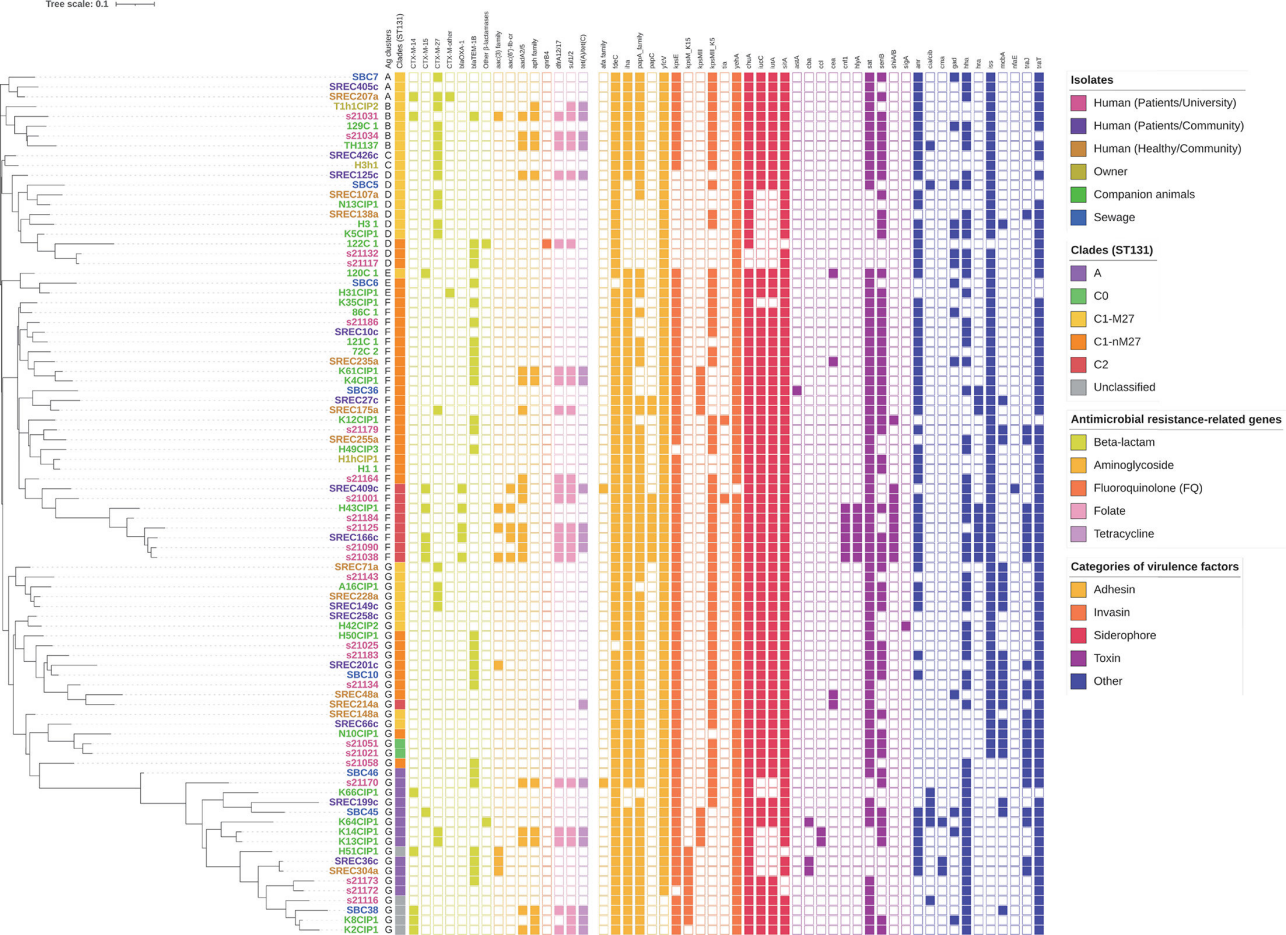
Accessory genome overview and presence of AMR-related genes and VF genes in 88 ST131 isolates. The phylogenetic tree represents relationships among ST131 isolates based on accessory genome analysis. Each row in the heatmap corresponds to a selected AMR or VF gene, and each column represents an individual isolate, arranged according to the tree. Colored indicates presence, and white indicates absence of the gene. Patterns suggest clade-associated differences in gene content. [AMR] CTX-M-other: including CTX-M-12, -13, -17, -24, -38, -55, -84, -102, -104, -125, -129, -130, -134, -192, and -196. CTX-M-55 was detected only in H31CIP1, whereas all other CTX-M variants in this group were exclusively found in SREC207a. Other β-lactamases: including CMY-2 and DHA-1. CMY-2 was detected only in K64CIP1, DHA-1 was detected only in 122C_1. aac(3) family: including *aac*(3)-IIa and *aac*(3)-IId. *aac*(3)-IIa was detected in three isolates (H43CIP1, s21038, and s21125), whereas *aac*(3)-IId was detected in the remaining isolates. aadA2/5: *aadA2* was detected only in s21001, whereas *aadA5* was detected in the remaining isolates. aph family: including *aph*(3'')-Ib and *aph*(6)-Id. Both were detected in all such isolates. *dfrA12*/*17: dfrA12* was detected only in s21001, whereas *dfrA17* was detected in the remaining isolates. sul1/2: *sul1* was detected in nine isolates (122C_1, SREC166c, SREC175a, SREC409c, s21001, s21038, and s21090, s21125, s21164), *sul2* was detected in two isolates (K8CIP1 and T1h1CIP2), whereas both were detected in the remaining isolates. tet(A)/tet(C): *tet*(C) was detected only in SREC214a, whereas *tet*(A) was detected in the remaining isolates. [VF] Twelve VF genes (*fimH*, *aslA*, *ompT*, *yehB*, *yehC*, *yehD*, *fyuA*, *irp2*, *csgA*, *nlpI*, *terC*, and *usp*) were detected in 100% of 88 isolates and are not shown in this heatmap. afa family: including *afaA*, *afaB*, *afaC*, *afaD*, and *afaE. afaA*, *afaC*, and *afaD* were detected in two isolates (SREC409c and s21170), whereas *afaB* and *afaE* were detected only in s21170. papA family: including *papA_F19*, *papA_F20*, *papA_F43*, *papA_F48*, *papA_F7-1*, *papA_F19*, and *papA_F20* were detected only in SREC235a, *papA_F48* was detected in two isolates (SREC27c and SREC175a), *papA_F7-1* was detected s21001 with *papA_F43*, whereas *papA_F43* was detected in the remaining isolates. shiA/B: *shiA* was detected in two isolates (K12CIP1 and SREC409c), whereas *shiB* was detected in seven isolates (s21001, H43CIP1, s21184, s21125, SREC166c, s21090, s21038). cia/cib: *cia* was detected in five isolates (TH1137, SBC5, SBC45, K64CIP1, s21116), whereas *cib* was detected in three isolates (K66CIP1, SREC199c, K64CIP1).

To investigate the genomic relationships among ST131 isolates from multiple sources, accessory genome analysis was performed on 88 ST131. This analysis divided the ST131 isolates into seven major accessory genome clusters, ranging in sizes from 2 to 38 isolates (Ag clusters A–G and Fig. 3). However, these clusters may reflect not only clonal relationships but also the influence of horizontally acquired elements, such as plasmids. Although the composition of these Ag clusters appeared to be broadly consistent with the classification of ST131 clades, this relationship should be interpreted with caution, as accessory genome profiles can be influenced by mobile genetic elements.

Ag clusters A–C were exclusively composed of clade C1-M27 isolates. Ag clusters D–E consisted of C1-M27 and C1-nM27, whereas Ag cluster F contained isolates from clades C1-nM27 and C2. In addition, Ag cluster G comprised isolates from multiple ST131 clades, including A, C0, C1-M27, C1-nM27, C2, and unclassified isolates. Six of the seven Ag clusters, except Ag cluster A, contained isolates from multiple sources (humans, companion animals, and/or sewage). The two largest clusters, G (*n* = 38) and F (*n* = 27), included isolates from humans (university hospital, community clinics, and healthy humans), companion animals, and sewage.

The distribution of 36 AMR genes was structured by the Ag clusters. The ESBL gene *bla*_CTX-M-27_ was detected in 50%–100% of isolates in Ag clusters A–D. In contrast, the possession was much lower in Ag clusters F (3.7%) and G (15.8%) (Table S3a). These FQ-R *E. coli* isolates in Ag clusters F and G possessed other type of ESBL genes, *bla*_CTX-M-15_ and *bla*_CTX-M-14_, respectively.

Significant differences in the prevalence of 59 VF genes were likewise observed among the Ag clusters. Twelve VF genes (*fimH*, *aslA*, *ompT*, *yehB*, *yehC*, *yehD*, *fyuA*, *irp2*, *csgA*, *nlpI*, *terC*, and *usp*) were detected in 100% of ST131 isolates analyzed. The prevalence of several VF genes including *kpsE*, *iucC*/*iutA*, *sat*, and *iha* was significantly lower in Ag cluster D compared to Ag clusters F and G (all Padj < 0.05, Table S3b). In contrast, the P fimbriae-associated genes *papC* and *hra* were detected in 33.3% of isolates in Ag cluster F but were absent in Ag cluster G (Padj = 0.011). Furthermore, the serum resistance gene *iss* was detected in all Ag cluster F isolates (100%), a significantly higher proportion than in Ag cluster G (52.6%, Padj = 0.0026).

For comparison, AMR/VF gene prevalence was also analyzed by source category and by ST131 clades. No significant associations were observed between gene content and source of origin (all Padj > 0.05 and Table S3c and d). In contrast, significant associations were found between certain genes and specific ST131 clades, consistent with the clade composition of the Ag clusters (e.g., *bla*_CTX-M-27_ with C1-M27 and Table S3e; *papC* with C2 and Table S3f).

### Core genome SNP analysis of ST131 isolates obtained in this study

**Fig 4:**
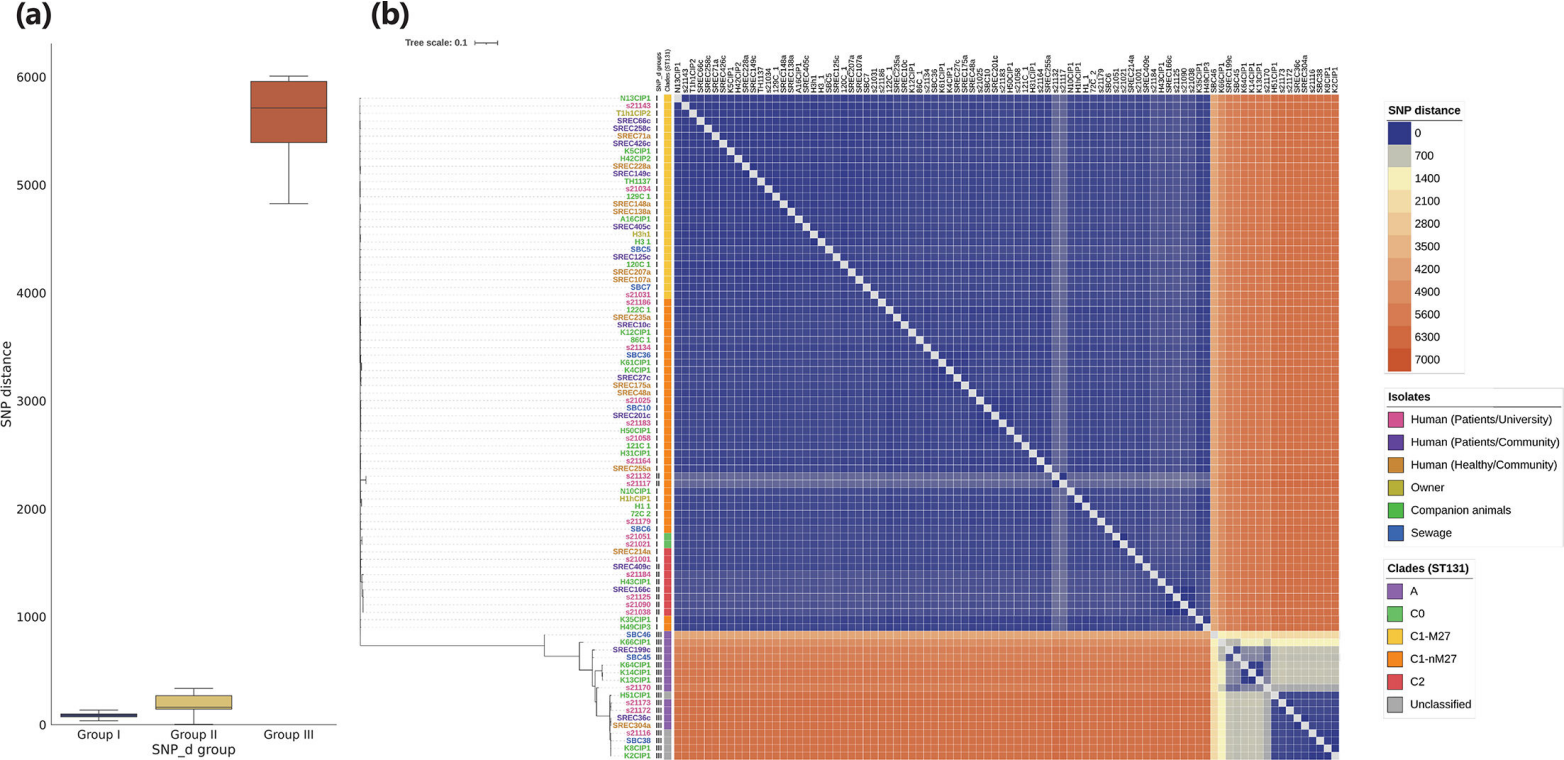
Core genome SNP analysis and clustering of 88 ST131 isolates. (**a**) Distribution of all 3,828 pairwise core genome SNP distances. Pairwise comparisons were classified into three SNP distance groups (SNP_d groups) based on SNP thresholds: I (<100 SNPs), II (100–300 SNPs), and III (>300 SNPs). (**b**) Maximum-likelihood phylogenetic tree based on 8,911 core genome SNPs. The SNP_d group (I–III) defined in panel a and ST131 clades are labeled next to each isolate name. The adjacent heatmap indicates pairwise SNP distances between isolates, with colors ranging from 0 SNPs (blue) to 7,000 SNPs (red). Detailed analysis of SNP_d group I is shown in Fig. 5.

Core genome SNP analysis of the 88 ST131 isolates identified a total of 8,911 core genome SNPs. We then performed all possible pairwise comparisons among the 88 isolates (*n* = 3,828), with each comparison representing a pair of isolates. Pairwise core genome SNP distances ranged from 1 to 6,141 SNPs. Based on these distances, we categorized the isolate pairs into three SNP distance groups (SNP_d groups) to investigate the relationships among closely related isolates (Fig. 4a). The SNP_d groups were defined as follows: I (SNPs < 100; 62 isolates), II (SNPs 100–300; 9 isolates), and III (SNPs > 300; 17 isolates).

A phylogenetic tree based on the core genome SNP analysis revealed that the clustering largely corresponded with SNP_d groups and the established ST131 clade classification (Fig. 4b). The first cluster (SNP_d groups I and II) consisted of clade C isolates (group I: C0, C1-M27, C1-nM27, and C2; group II: C1-nM27 and C2), whereas the second cluster (SNP_d group III) comprised non-C clades (clades A and unclassified). Notably, each cluster contained isolates from multiple sources, including humans, companion animals, and sewage.

**Fig 5:**
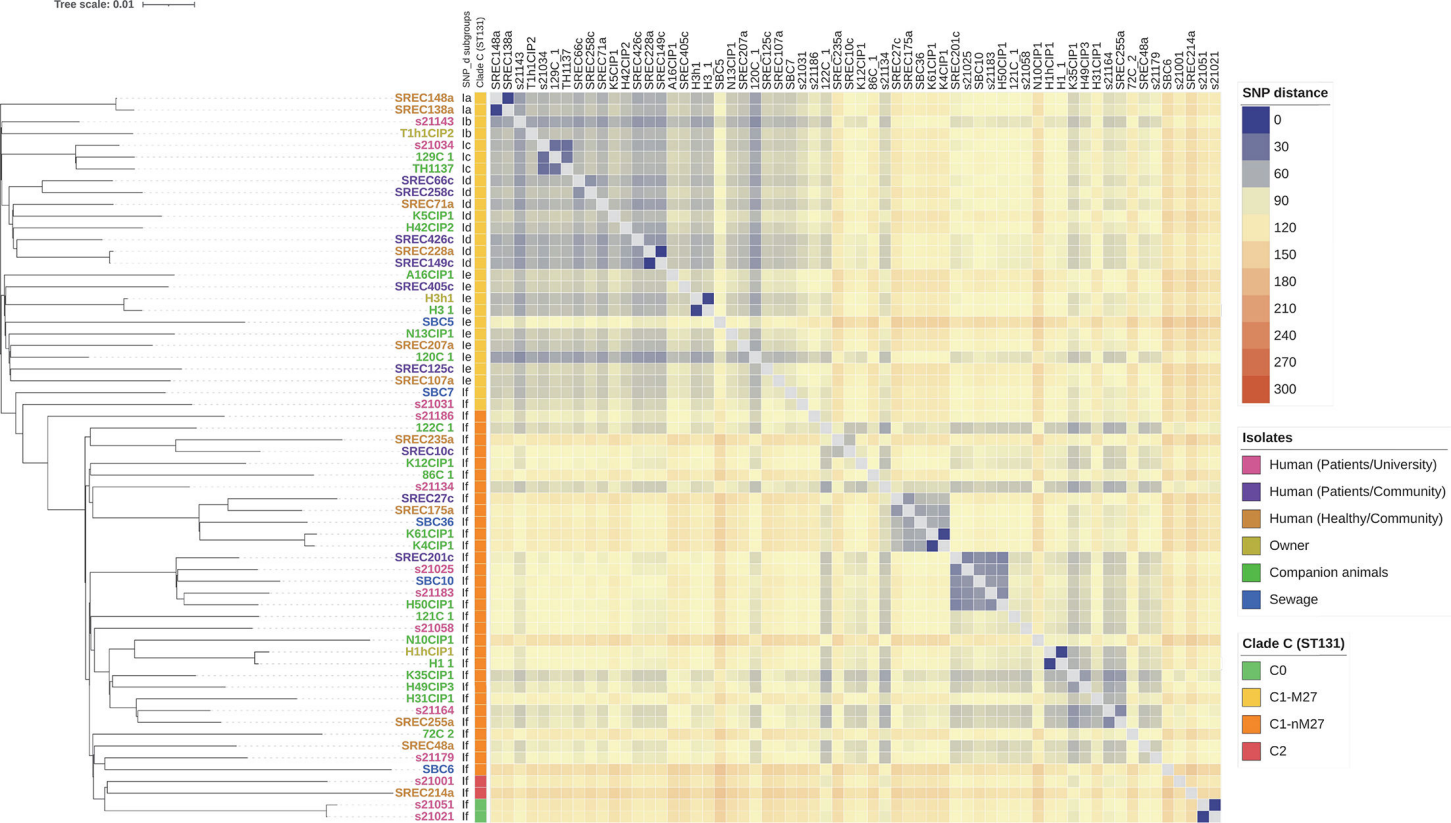
Detailed core genome SNP-based phylogenetic analysis of ST131 SNP_d group I. Detailed maximum-likelihood tree of 62 ST131 isolates within SNP_d group I (identified in Fig. 4a) was reconstructed based on 2,136 core genome SNPs, with subgroups (SNP_d group Ia–If), and clades are labeled next to each isolate name. The adjacent heatmap shows pairwise SNP differences, with colors ranging from 0 SNPs (blue) to 300 SNPs (red).

To investigate core genome relatedness in more detail, we constructed a core genome SNP-based phylogenetic tree for the 62 isolates belonging to SNP_d group I (Fig. 5). This analysis identified 2,136 core genome SNPs and further subdivided SNP_d group I into six subgroups (SNP_d subgroups Ia–If), each containing 2 to 37 isolates. Subgroups Ia and Ib consisted exclusively of human-derived isolates, whereas subgroups Ic and Id included isolates from both humans and companion animals. Subgroups Ie and If comprised isolates from humans, companion animals, and sewage. Pairwise core genome SNP distances within this subgroup ranged from 1 to 165 SNPs.

Among these, six isolate pairs exhibited extremely low pairwise SNP distances (1–6 SNPs), suggesting particularly close genetic relationships. These included three human–human pairs: a community–healthy pair (1 SNP; SREC149c vs SREC228a) and a healthy–healthy pair (5 SNPs; SREC138a vs SREC148a), both with unknown epidemiological link, and a university–university pair (3 SNPs; s21021 vs s21051) from the same university hospital. In addition, two companion animal–owner pairs (5 and 6 SNPs; H1_1 vs H1hCIP1 and H3_1 vs H3h1, respectively) were obtained from the same households, and one companion animal–companion animal pair (6 SNPs; K4CIP1 vs K61CIP1) was obtained from different households but the same small animal clinic.

### Genomic relatedness and accessory genome diversity in companion animal–owner pairs

**Fig 6:**
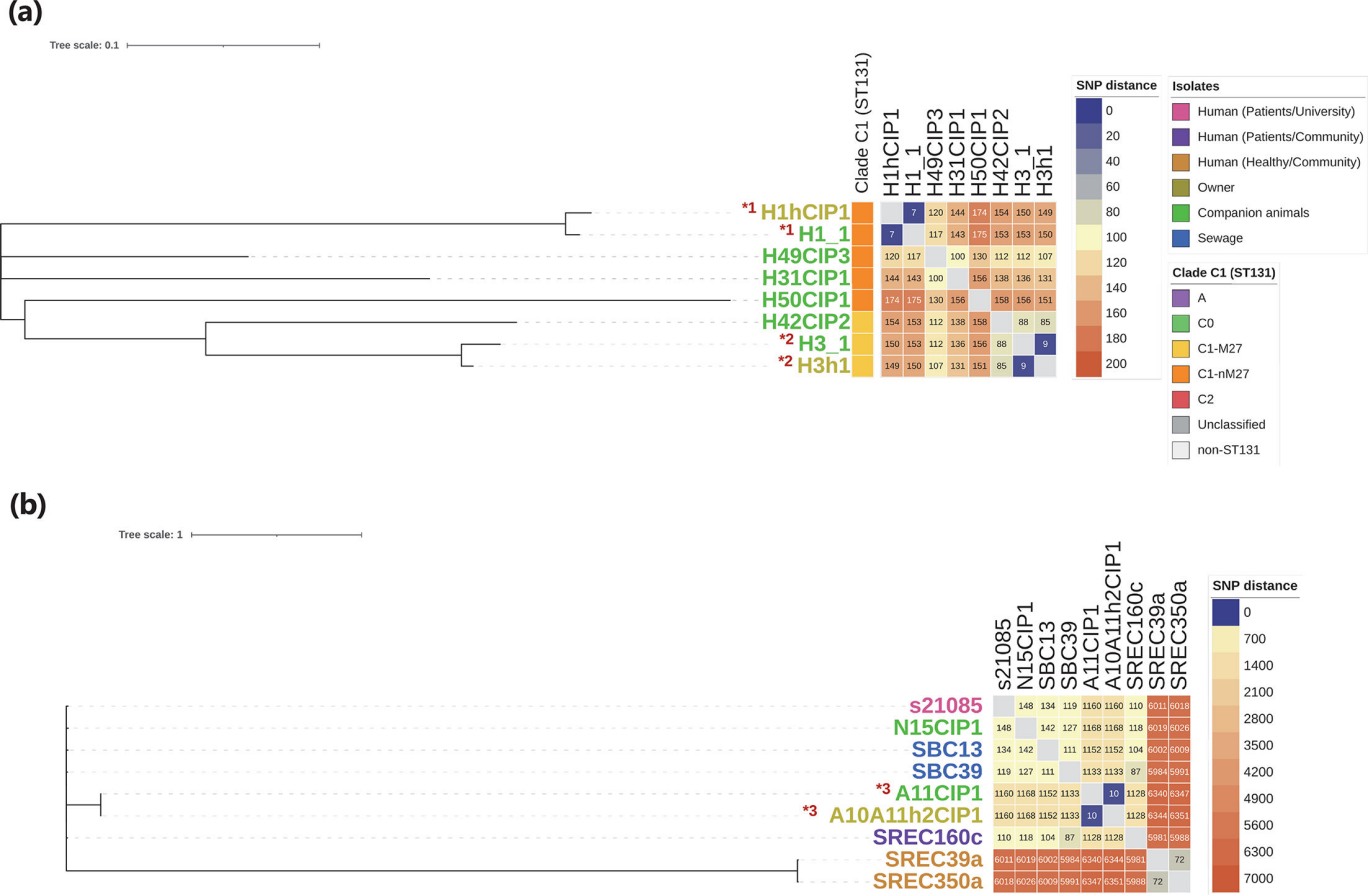
Core genome SNP-based phylogenetic trees and pairwise SNP distance heatmaps of three companion animal–owner pairs (Pair 1–Pair 3). (**a**) Comparison of ST131 clade C1 isolates from two companion animal–owner pairs (indicated by asterisks) with four other clade C1 isolates from companion animals visiting the same small animal clinic. This analysis was based on 388 core genome SNPs. ^*1^: Pair 1 (H1_1/H1hCIP1); ^*2^: Pair 2 (H3_1/H3h1). (**b**) Comparison of isolates from the ST38 companion animal–owner pair (^*3^: Pair 3, A10A11h2CIP1/A11CIP1) with seven other ST38 isolates from diverse sources. This analysis was based on 7,021 core genome SNPs. Phylogenetic trees based on core genome SNPs are shown on the left, and corresponding heatmaps of pairwise SNP distances are shown on the right. Heatmap cells are annotated with SNP values, and colors indicate pairwise SNP distances, ranging from blue (fewer SNPs) to red (more SNPs).

Among the five companion animal–owner pairs, FQ-R *E. coli* isolates were recovered from both the owner(s) and the animal in four pairs, and these pairs shared the same STs (Pairs 1–4; Table 2). To evaluate within-household genetic relatedness and assess pair-specific differences, we conducted comparative analyses of the core genome SNPs for ST131 clade C1 isolates (Pairs 1 and 2) and ST38 isolates (Pair 3) (Fig. 6a and b).

**TABLE 2:** Characteristics of the four companion animal–owner pairs and their FQ-R *E. coli* isolates

Pair	Strain	Host	ST	Clade
Pair 1	H1_1	Companion animal	131	C1-nM27
	H1hCIP1	Owner	131	C1-nM27
Pair 2	H3_1	Companion animal	131	C1-M27
	H3h1	Owner	131	C1-M27
Pair 3	A10CIP1	Companion animal	224	–^*a*^
	A11CIP1	Companion animal	38	–
	A10A11h2CIP1	Owner	38	–
Pair 4	T1CIP1	Companion animal	2380	–
	T1h1CIP2	Owner A	131	C1-M27
	T1h2CIP1	Owner B	2380	–

^*a*^ –, not tested.

In Pairs 1 and 2, both companion animals had visited the same small animal clinic. Therefore, we additionally compared these isolates with other ST131 clade C1 isolates obtained from the same clinic. Analysis of these 9 isolates identified a total of 388 core genome SNPs, with pairwise SNP distances ranging from 7 to 175 SNPs. In both pairs, the companion animal–owner isolates combinations exhibited the lowest SNP distances: 7 SNPs between H1_1 (dog) and H1hCIP1 (owner) in Pair 1 and 9 SNPs between H3_1 (dog) and H3h1 (owner) in Pair 2. In Pair 3, the companion animal–owner isolate combination was compared with seven additional ST38 isolates derived from diverse sources in Sapporo (two each from healthy humans and sewage, and one each from a university hospital, a community clinic, and a dog). Analysis of these 9 isolates identified a total of 7,021 core genome SNPs, with pairwise SNP distances ranging from 10 to 6,351 SNPs. Among these, the companion animal–owner combination again exhibited the lowest SNP distance (10 SNPs; A11CIP1 vs A10A11h2CIP1). In Pair 4 (ST2380), the SNP distance between T1CIP1 (dog) and T1h2CIP1 (the owner) was 24.

### Comparison of ST131 isolates from Sapporo and international collections based on core genome SNPs

To compare the genetic similarity of the 88 ST131 isolates obtained in this study with those from other countries, we performed a core genome SNP analysis incorporating 135 international ST131 genomes derived from 16 countries and diverse sources (humans, companion animals, water, and livestock), resulting in a combined data set of 223 isolates. This analysis identified a total of 10,381 core genome SNPs, and pairwise SNP distances ranged from 0 to 4,419 SNPs.

**Fig 7:**
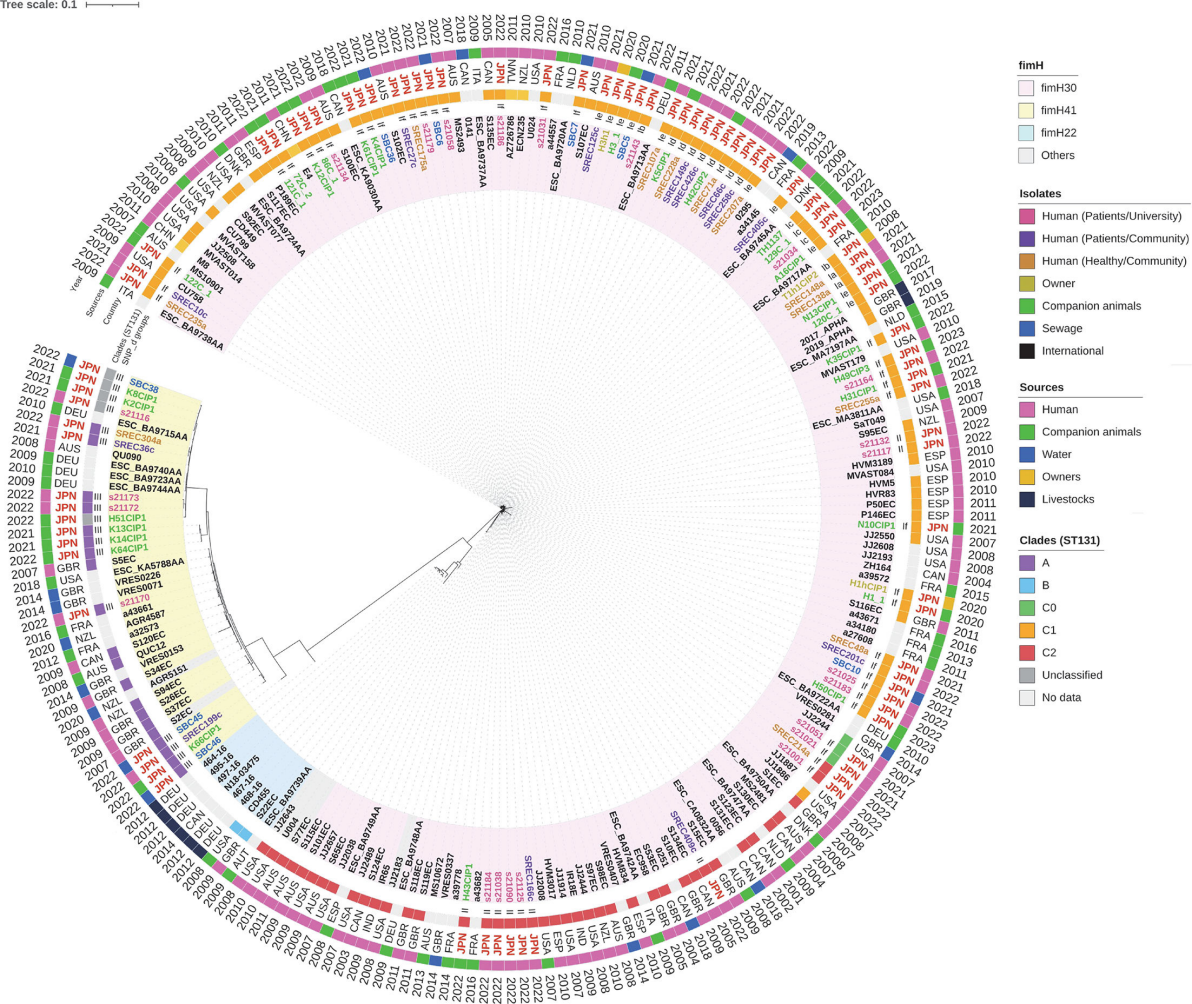
Core genome SNP-based phylogenetic analysis of 88 Sapporo isolates and 135 international ST131 isolates. Maximum-likelihood phylogenetic tree of the combined 223 isolates based on 10,381 core genome SNPs. *fimH* type, SNP_d groups, ST131 clades, country, sources, and year are labeled next to each isolate name. All isolates indicated as “JPN” in the country annotation correspond to the Sapporo isolates (this study). [Country] AUS: Australia, AUT: Austria, CAN: Canada, CHN: China, DNK: Denmark, FRA: France, DEU: Germany, IND: India, ITA: Italy, JPN: Japan, NLD: Netherlands, NZL: New Zealand, ESP: Spain, TWN: Taiwan, GBR: United Kingdom, USA: United States of America.

**Fig 8:**
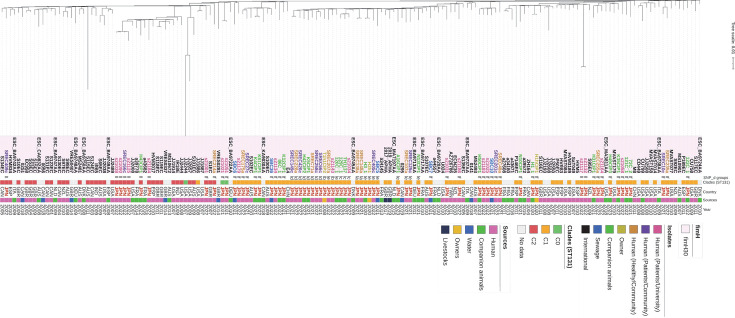
Detailed core genome SNP-based phylogenetic analysis of 172 ST131-*fimH*30 isolates. Maximum-likelihood phylogenetic tree of the 172 ST131-*fimH*30 isolates (a subset from Fig. 7) based on 4,661 core genome SNPs. Annotations (including *fimH* type, SNP_d groups, ST131 clades, country, sources, and year) are labeled as in Fig. 7. Isolates from Sapporo are indicated as “JPN” in the country annotation. [Country] AUS: Australia, CAN: Canada, CHN: China, DNK: Denmark, FRA: France, DEU: Germany, IND: India, ITA: Italy, JPN: Japan, NLD: Netherlands, NZL: New Zealand, ESP: Spain, TWN: Taiwan, GBR: United Kingdom, USA: United States of America.

The core genome SNP-based phylogeny revealed that the ST131 isolates in this study tended to co-cluster with international ST131 genomes in a manner largely dependent on *fimH* type (Fig. 7). In addition, the core genome SNP-based phylogeny of the 172 ST131-*fimH*30 isolates, which was based on a total of 4,661 core genome SNPs, showed that although the phylogeny was further divided into several clusters corresponding to the SNP_d groups, the international isolates were included within the respective clusters of our ST131 collection despite differences in their geographical origin and year of isolation (Fig. 8). Pairwise core genome SNP distances within this group ranged from 0 to 578 SNPs. These international ST131-*fimH*30 isolates were also predominantly identified from human and companion animals.

### Long-read sequencing of reveals shared ESBL plasmids among ST131 isolates

**Fig 9:**
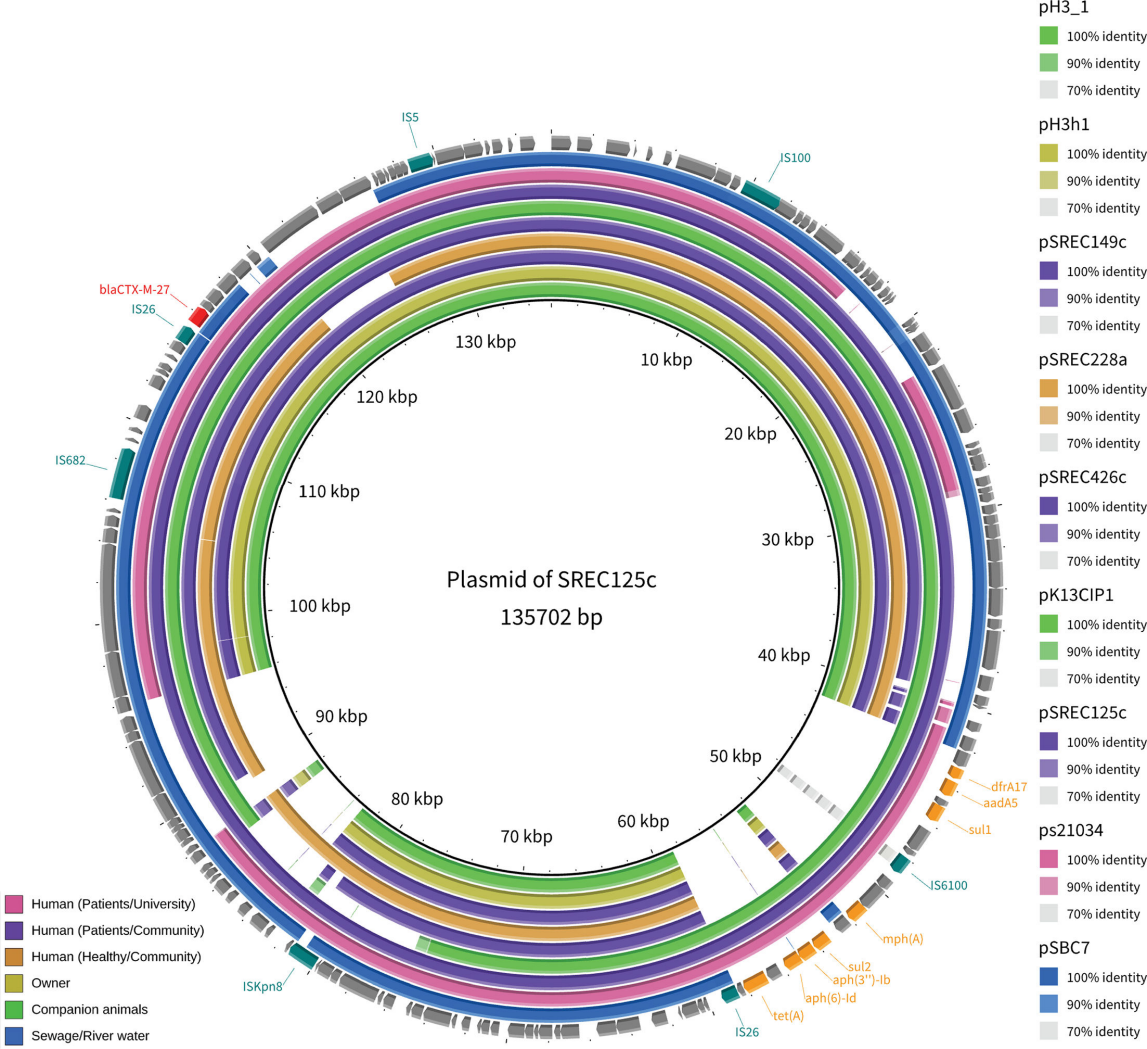
Comparative sequence analysis of *bla*_CTX-M-27_-harboring plasmids. The innermost black ring represents the reference sequence of pSREC125c (135,702 bp). Concentric colored rings indicate BLASTn sequence identity of the query plasmids against the reference. The rings are arranged from inner to outer, corresponding to the strain list from top to bottom in the right-side legend. Ring colors represent the sources of the isolates. The outermost circular map shows the positions of predicted open reading frames (ORFs) on the reference sequence; red arrows indicate *bla*_CTX-M-27_, orange arrows indicate other antimicrobial resistance genes, blue-green arrows indicate mobile genetic elements (insertion sequence, IS and transposon, Tn), and gray arrows indicate general coding sequences (CDS). The *bla*_CTX-M-27_ harboring plasmids were derived from patients at a university hospital (ps21034) and clinics (pSREC149c, pSREC426c, pSREC125c), a healthy human (pSREC228a), dogs (pH3_1, pK13CIP1), one dog owner (pH3h1, dog owner of H3_1), and sewage (pSBC7).

To further investigate the role of mobile genetic elements in the dissemination of ST131, we performed long-read sequencing on representative isolates carrying *bla*CTX-M genes (*n* = 9 and 3 for *bla*_CTX-M-27_ and *bla*_CTX-M-15_ harboring plasmids, respectively). Plasmid analysis revealed that *bla*_CTX-M-27_ and *bla*_CTX-M-15_ harboring plasmids exhibited highly similar backbone structures and nucleotide sequences across multiple isolates (Fig. 9 and Fig. S5). Among the nine *bla*_CTX-M-27_-harboring plasmids [from patients at a university hospital (*n* = 1) and clinics (*n* = 3), a healthy human (*n* = 1), dogs (*n* = 2), one dog owner (*n* = 1), and sewage (*n* = 1)], we observed high nucleotide sequence similarity between pH3_1 (from a dog) and pH3h1 (from the owner) (99.85%). Notably, pH3_1 also showed high sequence similarity to other *bla*_CTX-M-27_-harboring plasmids, including those from patients in community clinics, pSREC125c (99.92%), pSREC426c (97.92%), and pSREC149c (95.26%), as well as a plasmid from a dog (pK13CIP1, 93.25%) and from a university hospital patient (ps21034, 83.71%). Among the three *bla*_CTX-M-15_-harboring plasmids, nucleotide sequence similarity was 56.77% with ps21038 (from a university hospital patient) and 71.55% with pSREC166c (from a community clinic patient) when using pSREC409c (from a community clinic patient) as the reference.

We also analyzed the structure of *bla*_CTX-M-27_ and *bla*_CTX-M-15_ surrounding regions.

**Fig 10:**
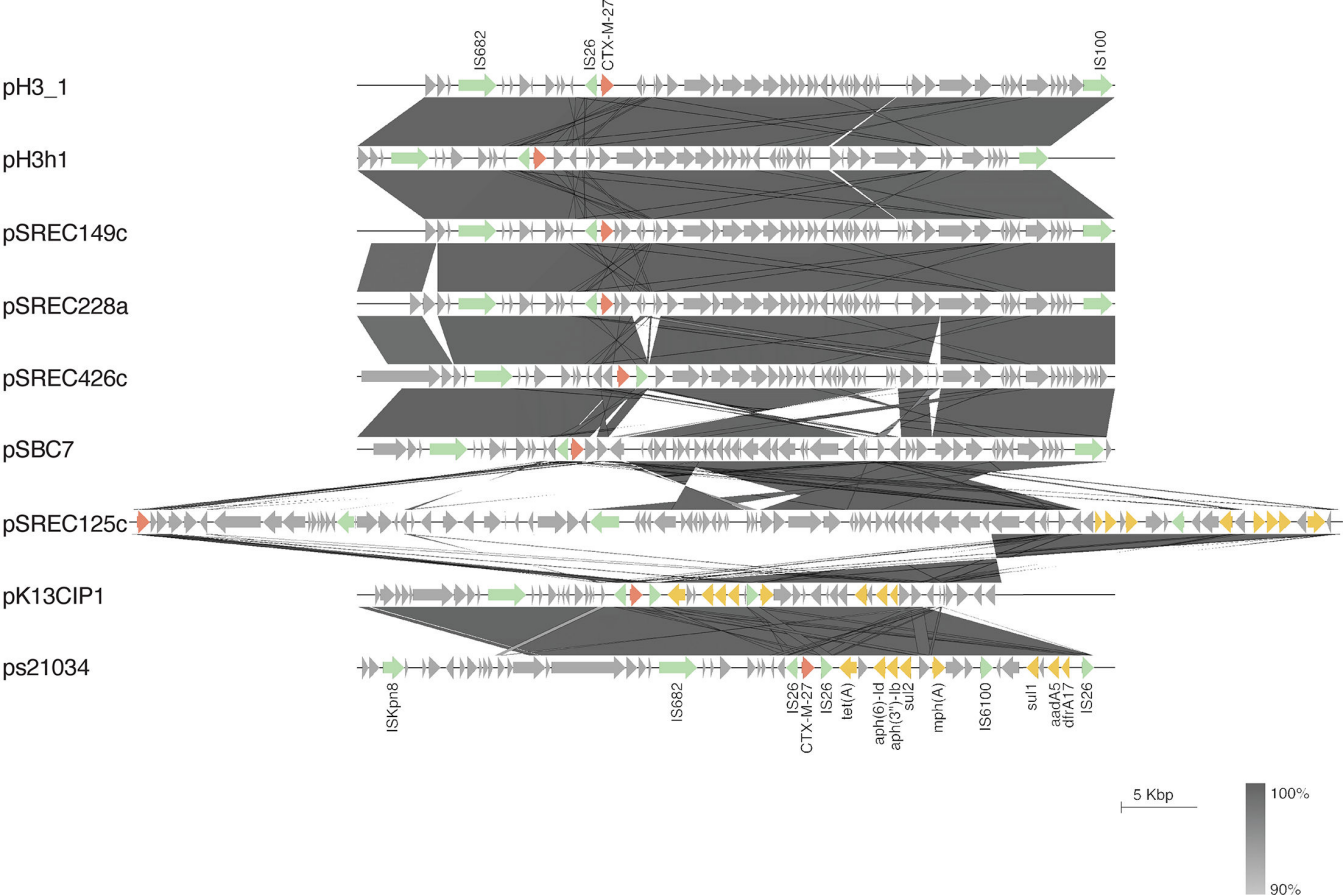
Linear sequence comparison of the *bla*_CTX-M-27_ genetic context. Arrows indicate coding sequences (CDS). Red arrows indicate *bla*_CTX-M-27_. Yellow arrows indicate other antimicrobial resistance genes. Green arrows indicate mobile genetic elements (insertion sequence, IS and transposon, Tn), and gray arrows indicate other CDS. Gray shading between sequences reflects BLASTn nucleotide identity of >90%. The *bla*_CTX-M-27_ harboring plasmids were derived from patients at a university hospital (ps21034) and clinics (pSREC149c, pSREC426c, pSREC125c), a healthy human (pSREC228a), dogs (pH3_1, pK13CIP1), one dog owner (pH3h1, dog owner of H3_1), and sewage (pSBC7).

While structural variations, including insertions, deletions, and inversions, were observed among the plasmids, the overall genetic backbone remained conserved, particularly in the *bla*_CTX-M_-associated regions, which were consistently flanked by insertion sequences such as IS100, IS26, and IS682 in *bla*_CTX-M-27_-harboring plasmids, IS6100, and IS26 (Fig. 10 and Fig. S6). The *bla*_CTX-M15_ was not located on the two plasmids (ps21038 and pSREC166c) but was instead integrated into the chromosomes (Chr. s21038 and Chr. SREC166c) (Fig. S6)

## DISCUSSION

Previous large-scale genomic analyses of FQ-R *E. coli* have been reported; however, most relied on publicly available genomes or isolates lacking clear epidemiological links, such as those collected across broad geographic areas or extended time periods (5, 20–26), and often lacked a unified sampling design based on the One Health concept (5, 21–23). In contrast, studies incorporating rigorously controlled sampling have been limited in both scale and analytical depth (20, 24–26).

In this study, we addressed these limitations by constructing a systematically designed research model under the One Health framework that unified sampling both spatially (within a single community level) and temporally (within a relatively short two-year period). This rigorous and structured approach (community- and household-integrated One Health approach) enabled us to clarify local dissemination dynamics of AMR pandemic clones with unprecedented resolution, generating data of high reliability and validity. Our findings provide crucial scientific evidence for understanding how AMR clones spread within communities, expand across regions, and ultimately establish global pandemics. Specifically, we elucidated community-level dissemination of FQ-R *E. coli* and demonstrated that particular lineages, such as ST131 and ST1193, have circulated among humans and companion animals within a defined community.

Within the ST131 population, multiple distinct clusters were identified based on accessory genome composition and core genome SNPs, suggesting that ST131 has disseminated at the community level through several co-circulating lineages. In particular, several ST131 lineages belonging to Ag clusters B–G in the accessory genome-based phylogeny and SNP_d group I in the core genome SNP analysis were primarily responsible for the major dissemination events within this regional model. Most of these isolates represented the classical clade C1 carrying *fimH*30. The predominant expansion of ST131 clade C1 and/or *fimH*30 observed in this study is consistent with reports from other regions in Japan and many other countries (6, 28, 29), indicating that the dissemination pattern demonstrated in the Sapporo area reflects the nationwide and global spread of ST131. These isolates originated from fecal samples of healthy humans and specimens from outpatients suggesting that a substantial proportion (5.6%–5.9%) of the general population carries ST131 asymptomatically. Additionally, these lineages included isolates recovered from patients with infections, predominantly urinary tract infections at a university hospital, supporting ST131 as a true etiological agent of clinical diseases and a contributor to hospital-associated problems including AMR. Although our study design represents a cross-sectional snapshot rather than a longitudinal survey, the frequent detection of ST131 in fecal samples from healthy individuals suggests that asymptomatic carriage may contribute substantially to its community-level persistence. Collectively, these observations indicate that asymptomatic carriage within human communities likely plays a major role in the ongoing dissemination of ST131. This interpretation is consistent with recent longitudinal household studies demonstrating persistent community reservoirs of ST131 (30).

Importantly, part of this dissemination appears to involve cross-host transmission between companion animals and their owners. This is supported by the extremely small SNP distances observed between companion animal-owner isolate pairs of ST131. Although the frequency of such household cross-host transmission events remains unclear, attention is warranted because ST131 is recognized as a major concern in human clinical settings carrying AMR genes including ESBL genes and VFs. This risk is also relevant to companion animals, as ST131 is a major ExPEC lineage responsible for urinary tract and bloodstream infections in dogs and cats (18). Household cross-transmission was further suggested in other FQ-R *E. coli* lineages, such as ST38 and ST2380 (10 and 24 SNP differences, respectively), indicating that such risks should be considered more broadly across *E. coli* populations. Thus, we need to focus on cross-host transmission events within households by collecting samples that include a sufficient number of companion animal–owner pairs.

In addition, our long-read sequencing data provide insights into the role of plasmids in the dissemination of ST131. We identified *bla*_CTX-M-27_- and *bla*_CTX-M-15_-carrying plasmids with varying degrees of sequence similarity across multiple isolates. In particular, *bla*_CTX-M-27_-harboring plasmids exhibited highly conserved backbone structures and near-identical sequences across isolates from both humans and companion animals, suggesting the circulation of closely related ESBL plasmids within and across reservoirs. In contrast, *bla*_CTX-M-15_-harboring plasmids showed more moderate similarity, indicating partial conservation and greater structural diversity. These findings suggest that ST131 dissemination is driven not only by clonal expansion but also by the spread of mobile genetic elements and that resistance determinants may be shared across intra-host and inter-reservoir settings within a One Health framework. However, this analysis was limited to representative isolates, and plasmid diversity and transmission dynamics may not have been fully captured. In addition, the directionality of plasmid transfer could not be determined. Further large-scale plasmid-resolved analyses are needed to clarify the role of plasmids in ST131 dissemination.

In our previous study, FQ-R *E. coli*, including ST131 and ST1193, were isolated from companion animals in Sapporo (5, 20, 27) and other regions of Japan (31–33). Isolation of ST131 from companion animals was not reported before 2009, but its occurrence has increased in recent years (31, 33). This trend, following the first isolation of ST131 in humans in 2001, suggests that its spread in humans may have partially triggered cross-host transmission between humans and companion animals, thereby contributing to the establishment and acceleration of the global ST131 pandemic.

A similar phenomenon may apply to the international FQ-R *E. coli* clone ST1193, which emerged and expanded rapidly among humans worldwide around the mid-2010s (34), and was subsequently isolated in companion animals in 2020 (34). In this study, we could not confirm direct transmission of ST1193 between humans and companion animals due to the small number of ST1193 isolates from companion animals (*n* = 5). Although 4 isolates were genetically distinct from human isolates, 1 isolate from a companion animal (K24CIP1) clustered with human isolates based on accessory genome and core genome SNP analyses, with pairwise SNP distances ranging from 53 to 111 (Fig. S4), suggesting that household cross-host transmission of ST1193 may also occur. Although it remains unclear whether ST1193 possesses traits enabling it to establish a stable pandemic comparable to ST131, our data showing that ST131 and ST1193 share similar accessory genome profiles based on COG categories such as those related to cell wall/membrane biogenesis, cell motility, defense mechanisms, and carbohydrate metabolism may support possibility. These findings suggest that the genomic repertoires of these pandemic lineages include advantageous traits promoting intestinal colonization and survival in both humans and companion animals, facilitating their dissemination within communities.

In this study, neither ST131 nor ST1193 was isolated from livestock. The absence or low frequency of these lineages in livestock and food products is consistent with previous studies (35, 36). A limitation of this study is the lack of sampling from food-chain products. Although further investigation is required to assess the spread of AMR *via* food-chain in this community, these observations support a limited contribution of food chain transmission *via* livestock compared with intra- and cross-host transmission among humans and companion animals. Furthermore, the isolation of ST131 and ST1193 in sewage samples, but not in river water, supports the notion that dissemination primarily occurs within human populations and that environmental spread remains limited. However, we recently reported the isolation of ST131 from wildlife and from river or lake water in other regions of Japan (12). These findings indicate that certain ST131 lineages may persist in non-human and non-companion animal hosts and warrants further attention. Additionally, phylogroup B1 lineages such as ST162 and ST533 were frequently isolated from livestock and river water, indicating the coexistence of ecologically adapted *E. coli* populations alongside human-associated populations within the same geographic region.

In conclusion, our systematically designed research model elucidated the community-level dissemination dynamics of the pandemic AMR clone *E. coli* ST131, demonstrating more clearly than ever before that its spread is driven primarily by human-to-human transmission and, to a lesser extent, by transmission between humans and companion animals. Importantly, several ST131 isolates obtained in this study clustered with isolates from other countries, indicating that the locally circulating lineages identified here are part of broader international transmission networks and that community-level dissemination can extend beyond regional and national boundaries. These findings strongly suggest the necessity of implementing community-level interventions in addition to current AMR control strategies, which mainly focus on hospital-based measures such as infection surveillance, antibiotic stewardship, and the prevention of nosocomial infections. Although this study did not quantify the frequency of FQ-R *E. coli* transmission between humans and their companion animals, further analyses involving a larger number of companion animal–owner pairs will be essential to determine its true extent. In addition, this study is limited by the lack of geographic mapping of isolate sampling locations due to privacy and ethical constraints, which prevented spatial analysis of transmission patterns. Incorporating such detailed epidemiological data in future studies would enable a more comprehensive understanding of ST131 dissemination dynamics. In Japan, the government has launched the Second National Action Plan on AMR (2023–2027), emphasizing the importance of AMR control under the One Health concept. Our findings not only provide scientific evidence supporting this framework but also clearly demonstrate the necessity of implementing a community- and household-integrated One Health approach as a key component of future AMR control strategies. This approach bridges human and animal health and offers a universal analytical model applicable to the investigation of other pandemic AMR clones.

## MATERIALS AND METHODS

### Sample origin

A total of 884 samples were collected from diverse sources in Sapporo and its suburb, Ebetsu, Japan, between 2021 and 2022. These included 651 human samples (215 from hospitalized patients at Sapporo Medical University Hospital, 220 from patients attending 40 community clinics, and 216 from healthy individuals), 85 samples from companion animals visiting 4 small animal clinics, 157 samples from livestock (108 cattle, 25 pigs, and 24 chickens) at 4 farms, 4 sewage samples, and 7 river water samples. Human clinical specimens included urine (*n* = 121), vaginal discharge (*n* = 32), pharyngeal swabs (*n* = 30), and other clinical specimens (*n* = 32). Fecal samples were collected from both community clinic patients (as part of routine clinical testing that may include cases with suspected foodborne illness or diarrhea) and healthy individuals (collected through general health checkups). Detailed information on sample sources and sample numbers is summarized in Table 3.

**TABLE 3:** Summary of sample sources and specimen types subjected in this study

Source	Subcategory	Specimen type	No. of samples (*n*)
Human	Hospitalized patients	Clinical specimens^*a*^ (urine, vaginal discharge, pharyngeal swab, others)	215
Human	Community clinic patients	Fecal samples	220
Human	Healthy individuals	Fecal samples	216
Companion animals	Dogs and cats	Rectal swabs	85
Livestock	Cattle	Rectal swabs	108
	Pigs	Rectal swabs	25
	Chicken	Rectal swabs	24
Environment	Sewage	Wastewater samples	4
	River water	Water samples (filtered)	7

^*a*^ Clinical specimens including urine (*n* = 121), vaginal discharge (*n* = 32), pharyngeal swab (*n* = 30), and other clinical specimens (*n* = 32). Other clinical specimens included respiratory specimens (nasal discharge, sputum, oral and tonsillar swabs; *n* = 10), skin and soft tissue specimens (*n* = 6), blood and ascitic fluid (*n* = 4), drain-related specimens (*n* = 5), gastrointestinal/biliary specimens (*n* = 4), and tissue biopsy or device-associated materials (*n* = 3).

### *E. coli* isolation

Isolation and identification of *E. coli* were performed using standardized procedures across all sample sources unless otherwise specified. Human clinical and fecal samples were streaked onto 5% sheep blood agar plates (Becton, Dickinson and Company, Franklin Lakes, NJ, USA) and incubated at 37°C overnight. Samples from animals and environmental sources were cultured on ECC-CHROM agar plates (Kanto Chemical Co., Inc., Tokyo, Japan) under the same incubation conditions. For environmental samples, sewage samples (500 mL each) were serially diluted in phosphate-buffered saline (PBS) up to 10⁻³, and 100 μL of each dilution was plated. River water samples (1 L each) were filtered through a 0.2 µm membrane filter (ADVANTEC, Tokyo, Japan), followed by resuspension in PBS, centrifugation (6,000 × *g*, 10 min), and plating after dilution. After overnight incubation at 37°C, up to three representative colonies per sample were selected for animal-derived samples, while up to 96 colonies per sample were screened for environmental samples. For human samples, a single colony per sample was selected as part of routine clinical diagnosis or investigation.

Selective isolation of FQ-R *E. coli* was performed among samples from companion animals, livestock, and environments. Samples as afordomention were also cultured on ECC-CHROM agar supplemented with 1 mg/L ciprofloxacin (Tokyo Chemical Industry Co., Ltd., Tokyo, Japan). For animal-derived samples, rectal swabs were first enriched in 3–5 mL Tryptic Soy Broth (TSB; Becton, Dickinson and Company, Franklin Lakes, NJ, USA) at 37°C overnight prior to plating. Thirty-eight FQ-R *E. coli* isolates derived from companion animals (32 dogs and 6 cats), collected during the same period (2021–2022) in Sapporo, were included based on previous studies (19, 26). Furthermore, 6 FQ-R *E. coli* isolates obtained from fecal samples of companion animal owners were also included in this study.

### *E. coli* identification and antimicrobial susceptibility testing

Suspected *E. coli* colonies were subcultured for confirmation and identified using matrix-assisted laser desorption/ionization time-of-flight mass spectrometry (MALDI-TOF-MS; Bruker Daltonics, Billerica, MA, USA). Antimicrobial susceptibility testing was performed using the microbroth dilution method according to Clinical and Laboratory Standards Institute (CLSI) guidelines, with *E. coli* ATCC 25922 used as the quality control strain.

### Molecular epidemiological analysis of FQ-R *E. coli* isolates

A total of 235 FQ-R *E. coli* isolates were included in the molecular epidemiological analyses. These isolates were derived from university hospital patients (*n* = 37), community clinics’ patients (*n* = 28), healthy humans (*n* = 31), companion animals (*n* = 55), owners (*n* = 6), sewage (*n* = 49), river water (*n* = 6), and livestock (cattle: *n* = 10, pigs: *n* = 1, chickens: *n* = 12).

ST131 and its clades (A, B, C, Unclassified), including subclades within clade C (C0, C1-M27, C1-nM27, and C2), were identified using multiplex PCR (37). WGS was then performed to characterize additional molecular epidemiological features of the 235 FQ-R *E. coli* isolates. Genomic DNA was extracted using the DNeasy Blood and Tissue Kit (QIAGEN, Hilden, Germany) according to the manufacturer’s protocol. The DNA libraries were prepared with the xGen DNA Library Prep Kit EZ UNI (Integrated DNA Technologies, Coralville, USA). Sequencing was conducted using the Illumina NovaSeq 6000 platform (Illumina, San Diego, USA). The resulting reads were de-multiplexed, quality-trimmed, and *de novo* assembled using CLC Genomics Workbench (QIAGEN).

Phylogenetic characteristics of the *E. coli* isolates were determined using *In Silico* Clermont Phylotyper (38). Multilocus sequence typing (MLST), *fimH* typing, and the detection of acquired antimicrobial resistance genes and VFs were performed using tools provided by the Center for Genomic Epidemiology: MLST 2.0, FimTyper 1.0, ResFinder 4.6.0, and VirulenceFinder 2.0 (39).

### Genome annotation, pangenome, and accessory genome analysis

Pangenome analysis was performed on 235 FQ-R *E. coli* isolates obtained in this study. Accessory genome analysis was conducted on the 88 ST131 isolates. Genomes were annotated using Prokka (v1.14.6) with default settings, and the resulting GFF files were used as input for pangenome analysis (40). Roary (v3.12.0) was applied to define the pangenome, with core genes defined as those present in ≥99% of isolates (41). Multiple sequence alignments of core genes were generated using MAFFT, and phylogenetic trees were constructed with FastTree under the generalized time-reversible (GTR) model. A separate phylogenetic tree based on the presence or absence of accessory genes was also generated. All trees were visualized using iTOL (Interactive Tree of Life) (42).

Genes present in at least 70% of all isolates were considered for downstream association analysis. Candidate genes were prioritized using Scoary2 based on odds ratios, *P*-values, multiple testing correction, and the Youden index to select genes most strongly associated with each ST (43). These metrics were chosen to identify genes that were both highly specific and consistently associated with each clone, facilitating robust functional comparisons in downstream analyses. Functional annotation of candidate genes was performed using EggNOG-mapper (v2.1.13), and COG categories were compared across STs to assess functional biases (44).

### Core genome SNP detection

Core genome SNP detection was performed for three datasets: (i) the 88 ST131 isolates obtained in this study, (ii) these isolates combined with 135 publicly available *E. coli* ST131 genomes with epidemiological information (origin, year of isolation, and country) available in the NCBI database (Table S4), and (iii) two companion animal–owner pair subsets (one consisting of ST38 isolates including pair A11CIP1/A10A11h2CIP1 and related strains from this study, and another consisting of ST131 clade C1 isolates collected from the same animal clinic, including pairs H3_1/H3h1 [C1-M27] and H1_1/H1hCIP1 [C1-nM27]. Publicly available genomes were selected from previously published studies deposited in NCBI in which ST131 designation and epidemiological metadata were clearly described. To enhance comparability with our data set, isolates were chosen to avoid substantial overrepresentation of specific sources or clades when such information was available and to include geographically diverse strains. The detection of core genome SNP was conducted using kSNP4 (45). The optimal k-mer size was determined for each data set using the built-in kchooser utility, and *k* = 17 was selected in all cases. For 135 publicly available genomes, raw reads (SRA) were *de novo* assembled with CLC Genomics Workbench (QIAGEN), and complete genomes (GenBank) were obtained as FASTA files. The reference genome used was *E. coli* strain EC958 (accession no. HG941718.1). Phylogenetic reconstruction was conducted using the maximum likelihood (ML) method, which estimates the most likely tree topology based on a nucleotide substitution model and is considered appropriate for genome-scale SNP analysis. The resulting tree was visualized with iTOL. Pairwise SNP distances were calculated using snp-dists (v0.8.2), and the resulting matrix was visualized as a heatmap using SRplot (46, 47).

### Long-read sequencing for plasmid analysis

To completely resolve plasmid structures and investigate horizontal gene transfer dynamics, long-read sequencing was performed on selected representative *E. coli* isolates based on phylogenetic clustering. High-molecular-weight genomic DNA was extracted using either the QIAGEN Genomic-tip (QIAGEN) or the Quick-DNA HMW MagBead Kit (Zymo Research, Irvine, CA, USA). Long-read libraries were prepared using the Rapid Barcoding Sequencing Kit SQK-RBK114.24 (Oxford Nanopore Technologies, Oxford, UK) and sequenced on a MinION device equipped with a FLO-MIN114 (R10.4.1) flow cell. Following basecalling, adapter trimming was performed using Porechop v0.2.4 (48), followed by quality filtered using Filtlong v0.2.1 (minimum length, 1,000 bp) (49). *De novo* genome assembly was conducted using Flye v2.9.6 (50) and Unicycler v0.5.1 with illumina short-read sequencing (51). Genome annotation was conducted using Prokka v1.14.6 (40), staramr v.0.12.1 (52), and MobileElementFinder v1.0.3 (53). Comparative genomic analyses were performed using BLAST Ring Image Generator 0.95 (BRIG) (54) and Easyfig (55) to visualize their structural characteristics and genetic contexts. Plasmid sequence similarity was calculated using dnadiff in the MUMmer v 4.0.0 based on aligned bases and average nucleotide identity (56).

### Statistical analysis

Comparative analyses of categorical data were performed using the chi-square test or Fisher’s exact test, as appropriate. For multi-category contingency tables containing small cell counts or zero values, *P*-values were estimated using Monte Carlo simulation (100,000 replicates). A *P*-value of less than 0.05 was considered indicative of statistical significance. *P*-values were adjusted for multiple testing by the Benjamini–Hochberg procedure to control the false discovery rate (FDR). An adjusted *P*-value (Padj) < 0.05 was considered statistically significant.

## Data Availability

The raw sequencing data of 200 FQ-R *E. coli* strains newly sequenced in this study were deposited in the NCBI Sequence Read Archive (SRA) under accession numbers SAMN52957538 to SAMN52957737 (BioProject number PRJNA1354839). The remaining 35 FQ-R *E. coli* strains analyzed in this study were previously reported and are available in the NCBI SRA under accession numbers SAMN48440591 to SAMN48440625 (BioProject number PRJNA1261911) (27).

## SUPPLEMENTAL MATERIAL

Fig. S1 aac.00007-26-s0001.tif Distribution of phylogenetic groups, STs, and ST131 clades of FQ-R *Escherichia coli* isolates by sources.

Fig. S2 aac.00007-26-s0002.tif Distribution of accessory genes across COG functional categories by ST.

Fig. S3 aac.00007-26-s0003.tif Functional enrichment of accessory genes by COG category and clone.

Fig. S4 aac.00007-26-s0004.tif Core genome SNP-based phylogenetic analysis of 40 ST1193 isolates.

Fig. S5 aac.00007-26-s0005.tif Comparative sequence analysis of *bla*_CTX-M-15_-harboring plasmids.

Fig. S6 aac.00007-26-s0006.tif Linear sequence comparison of the *bla*_CTX-M-15_ genetic context.

Supplemental tables aac.00007-26-s0007.xlsx Tables S1 to S4.

ASM does not own the copyrights to Supplemental Material that may be linked to, or accessed through, an article. The authors have granted ASM a non-exclusive, world-wide license to publish the Supplemental Material files. Please contact the corresponding author directly for reuse.
